# Digitally Driven Interdisciplinary Smile Redesign in a Patient with Midline Deviation: A Case Report

**DOI:** 10.1055/s-0045-1812109

**Published:** 2025-10-22

**Authors:** Ruri Nur Alinda, Putu Indra, Ferdinand Hadinata, Made Dwiandri Satyaputra, Aulia Ayub, Ananto Ali Alhasyimi

**Affiliations:** 1 Department of Orthodontics, Faculty of Dentistry, Universitas Gadjah Mada, Yogyakarta, Indonesia; 2 Smile Cloud Dental Clinic, Surabaya, East Java, Indonesia; 3 Faculty of Dentistry, Petra Christian University, Surabaya, Jawa Timur, Indonesia

**Keywords:** 3D facial scan, midline deviation, crown lengthening, interdisciplinary dentistry, patient satisfaction

## Abstract

A 29-year-old woman with midline deviation (maxillary dental midline was shifted 4.3 mm to the right side) and unsightly anterior veneers placed by an unlicensed dental professional, resulting in poor esthetic outcomes. Clinical findings included incorrect occlusion, asymmetric gingival contours, and overcontoured veneers. A multidisciplinary treatment strategy was created to rectify midline deviation and restore esthetics. This included the expertise of orthodontists, periodontists, endodontists, and restorative professionals. The patient underwent a series of treatments, starting with orthodontic realignment to correct occlusion and midline deviation, followed by periodontal intervention to address asymmetric gingival contours, and finally, restorative procedures involving facially directed zirconia veneers. Posttreatment, the dental midline was realigned toward the facial flow line, significantly improving smile symmetry and occlusal function. The patient reported high satisfaction with the outcome, particularly regarding the harmony between her dental and facial features. This case highlights the effectiveness of a digitally driven, interdisciplinary approach to correct complex esthetic issues following poor dental restorations. Three-dimensional facial scanning, in combination with orthodontic, periodontal, endodontic, and restorative treatments, provided precise and predictable results, leading to a highly successful outcome and improved patient satisfaction.

## Introduction

Today's society's increasing fascination with cosmetic dentistry necessitates orthodontic treatment. In cosmetic dentistry, orthodontic treatment is a frequently done procedure. It is implemented to obtain an esthetically pleasing appearance and a healthy occlusion by aligning the teeth. 1 2 As patient expectations for esthetic outcomes rise, especially among adults, orthodontic treatment alone may not solve all esthetic concerns. Patients are increasingly pursuing results that extend beyond mere tooth alignment and functional correction, but also anticipating improvements in smile harmony that can be obtained with an interdisciplinary approach. 3 4

Smile harmony encompasses a multidimensional balance among teeth alignment, gingival contour, and facial aesthetics, which may be assessed at the dimension of the face in all three spatial planes (the relationships of teeth to each other, to soft tissues, and to facial characteristics/macroesthetic), the correlation of lips, teeth, and gingiva (miniesthetic), and the fine structures of dental and gingival esthetics (microesthetic). 5 6 Key smile element such as the smile arc, buccal corridor, dental component (size, shape, color, alignment, crown angulation, midline, arch symmetry), and gingival display play critical roles in esthetic perception. 5 Midline deviation is a significant parameter in microesthetic analysis. Typically evaluated by millimetric displacement from the facial midline, recent studies show that the perceived esthetics of midline deviation is also influenced by the direction of deviation in relation to the facial flow line. Displacements toward the side of natural facial asymmetry designated as the “green zone” are regarded as more acceptable than those in the contrary direction “red zone,” even with a deviation of up to 4 mm. 7 The proportional width and height of anterior teeth, including the application of the golden ratio and the apparent width proportions also impact esthetic judgement. 5 6 Therefore, to attain long-term, harmonious smile outcomes, it is necessary to take a comprehensive and individualized strategy with an interdisciplinary approach between orthodontists, prosthodontists, and periodontists.

Recent advancements in digital dentistry have transformed the management of interdisciplinary approaches for esthetic situations. Digital technologies such three-dimensional (3D) facial scanning, digital smile design, intraoral scanning, and computer-aided design (CAD)/computer-aided manufacturing (CAM) systems have helped clinician to plan and perform restorative treatment with remarkable accuracy and reliability. 8 This digital approach enables clinicians visualize the final outcome prior to treatment initiation, enhancing communication among specialists and aligning the treatment plan with the patient's facial characteristics and esthetic preferences. Dental software helps with digital planning, visualization, treatment order, and exact execution of surgical and restorative operations. 9 10

Facial asymmetry and midline deviation are typically addressed through surgical intervention, with 3D virtual planning employed to enhance precision in outcomes. 11 This case report demonstrated how a patient who had orthodontic treatment improved their smile using a complete digital process that involved 3D facial scanning, intraoral scanning, and CAD/CAM zirconia veneers to create a personalized smile. This case report highlighted a novel nonsurgical, comprehensive facial-driven strategy to address the increasing cosmetic demand through the integration of digital technologies in interdisciplinary treatment.

## Case Report

### Case Presentation

**Fig. 1:**
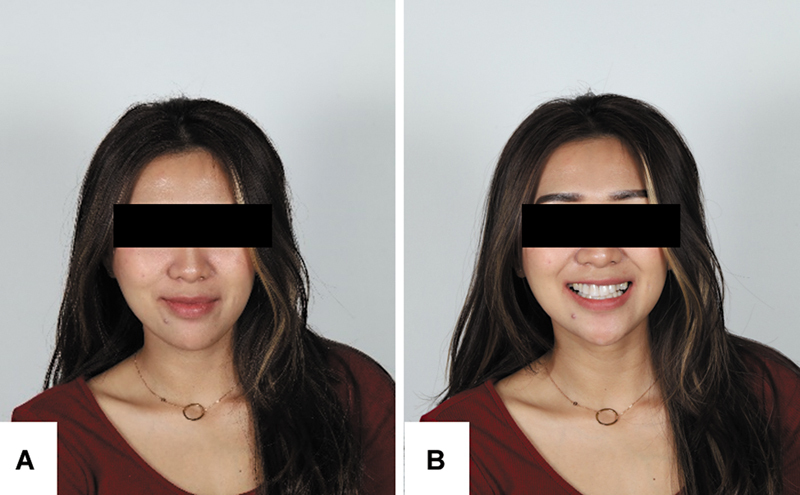
Pretreatment extraoral photographs. ( **A** ) Resting position. ( **B** ) Smile.

**Fig. 2:**
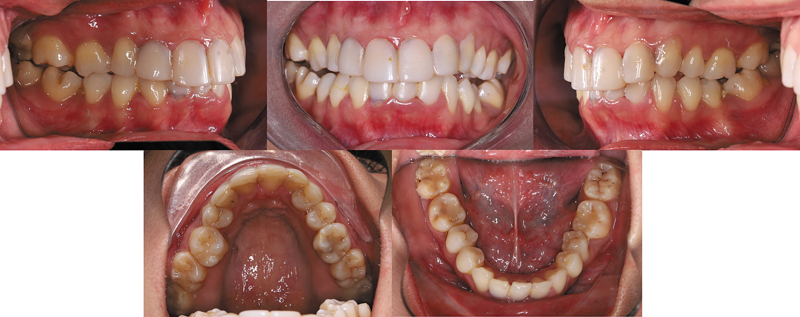
Pretreatment intraoral photographs.

**Fig. 3:**
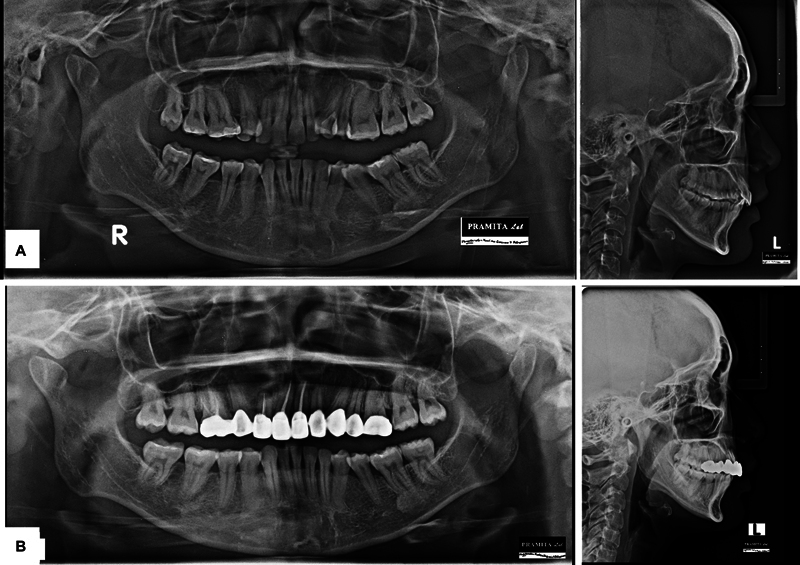
( **A** ) Pretreatment panoramic radiograph and lateral cephalogram. ( **B** ) Posttreatment panoramic and lateral cephalogram.

The patient provided written informed consent for the publication of this case report and the associated photographs. This case report was prepared in accordance with the CARE (CAse REport) guidelines. A 29-year-old female patient presented to the clinic with the chief complaint of unsatisfactory esthetics due to previous veneer placement by an unlicensed dental professional. She expressed the desire to improve the appearance and function of her anterior teeth and report dissatisfaction with the existing veneer, describing them as bulky and unnatural. Extraoral examination revealed a noticeable occlusal cant and slight facial asymmetry ( Fig. 1 ). Intraoral examination showed maxillary dental midline was shifted 4.3 mm to the right side, cups-to-cups relationship, missing teeth maxillary canines (teeth #13 and #23), and mandibular central incisor (teeth #41). The molar relationship revealed class II occlusion on both side, and canine class II on the left side. The overjet measured 3.4 mm, and the overbite was 3.8 mm. Patient also exhibited a constricted maxillary arch. Previously placed veneers on the anterior teeth (teeth #12, #11, #21, and #22) appeared overcontoured and poorly adapted ( Fig. 2 ). Cephalometric analysis indicated a skeletal class II relationship (A point-Nasion-B point = 7.33 degrees) with normal maxilla (Sella-Nasion-A point = 82.51 degrees) and retrognathic mandible (Sella-Nasion-B point = 75.17 degrees), hyperdivergent growth pattern ( Fig. 3 ). The patient exhibited good health, with no prior history of medical conditions. The treatment objective was to correct dental midline deviation, achieve optimal occlusion, maintain facial balance, and improve dental and facial esthetics.

### Case Management

In the beginning of the interdisciplinary treatment plan, orthodontic correction was performed with the utilization of a self-ligating fixed appliance system (Protect PT K; Protect Orthodontics, Zhejiang, China). Both the midline deviation and the occlusal cant were resolved by the utilization of unilateral posterior segmental distalization using the assistance of temporary anchorage devices (TADs) that were positioned in the left posterior maxilla.

After achieving optimal tooth position and occlusion, a postorthodontic esthetic digital workflow was commenced. This procedure encompassed digital facial scanning, intraoral scanning, periodontal crown lengthening, endodontic therapy, and final esthetic rehabilitation using facially directed CAD/CAM zirconia veneers on teeth 14 to 24.

**Fig. 4:**
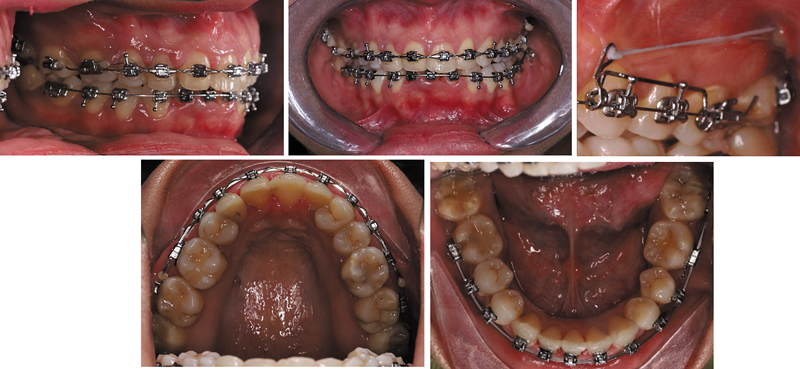
Intraoral photograph during treatment phase using self-ligating appliance and temporary anchorage device.

**Fig. 5:**
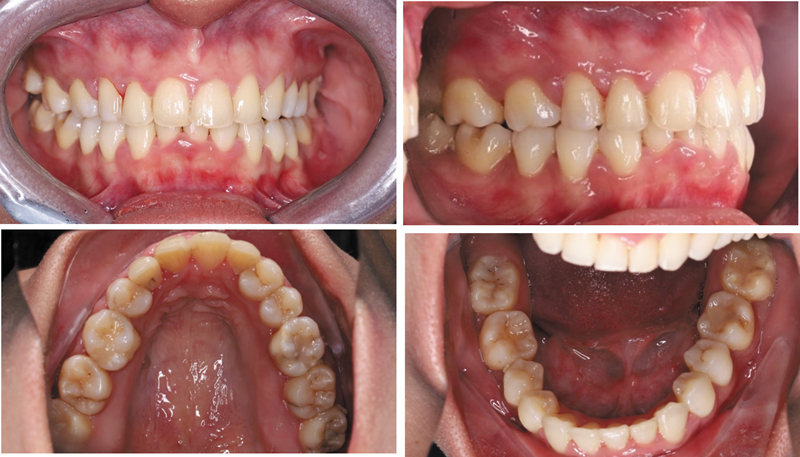
Postdebonding intraoral photographs.

Orthodontic treatment was performed over 11 months. High torque brackets were applied to teeth #21 and #22 and standard torque to teeth #11 and #12. TADs (Jeil Medical Corporation, Seoul, Korea: diameter 2 mm, length 12 mm) were placed for skeletal anchorage ( Fig. 4 ). Segmental distalization was used to close the space and correct midline discrepancies. A favorable occlusal relationship was obtained at the conclusion of the treatment, as evidenced by the correction of occlusal plane canting, the coincidence of upper dental midlines with the upper jaw, and the attainment of canine class I ( Fig. 5 ).

**Fig. 6:**
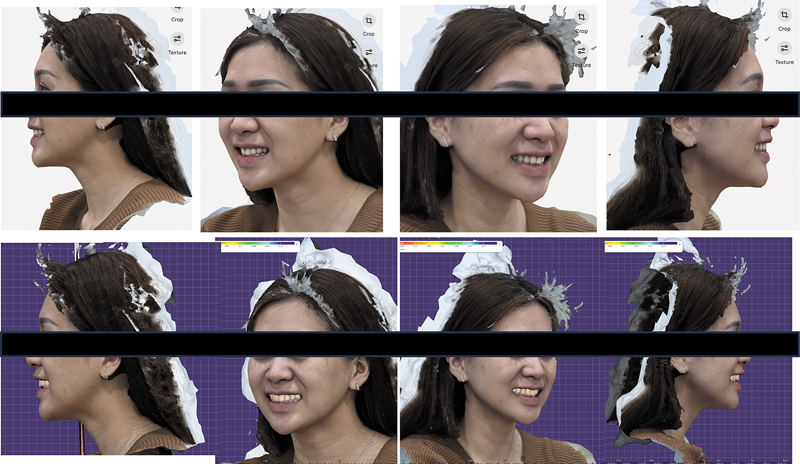
Right and lateral views from a three-dimensional (3D) facial scan.

Following debonding, a 3D facial scan and intraoral scan was conducted to assess smile esthetics in relation to face features. The digital smile analysis was then conducted using a dental design software program (Exocad dental CAD; Exocad GmbH, Germany) ( Fig. 6 ). Crown lengthening was performed on the gingiva of teeth #14 to #24 to provide optimal gingival contouring using a temporary crown as diagnostic and esthetic guide. A mock-up was placed based on the planned design and evaluated over a period of 7 days. The result showed that anterior contacts were stable and functionally safe with no sign of premature contact or fracture. The cervical soft tissue exhibited health contours with no sign of inflammation indicating good biocompatibility and proper marginal adaption of the temporary restorations.

**Fig. 7:**
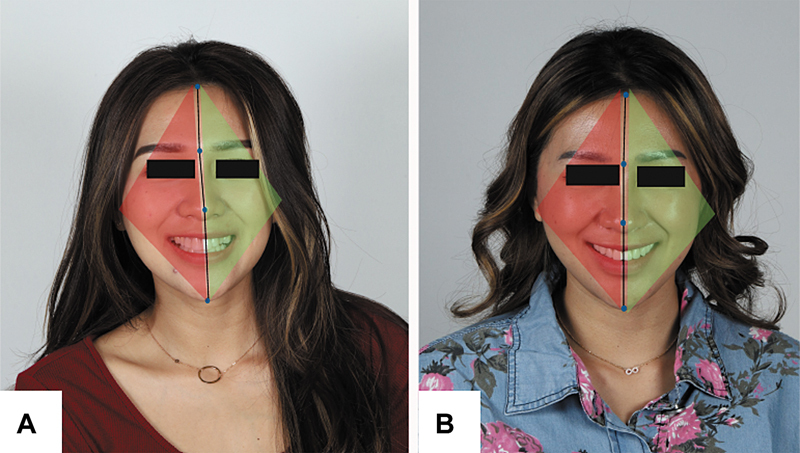
( **A** ) Pretreatment frontal smile showing dental midline deviation toward the red zone. ( **B** ) Posttreatment image demonstrating midline correction into the green zone, aligned with the facial flow line.

**Fig. 8:**
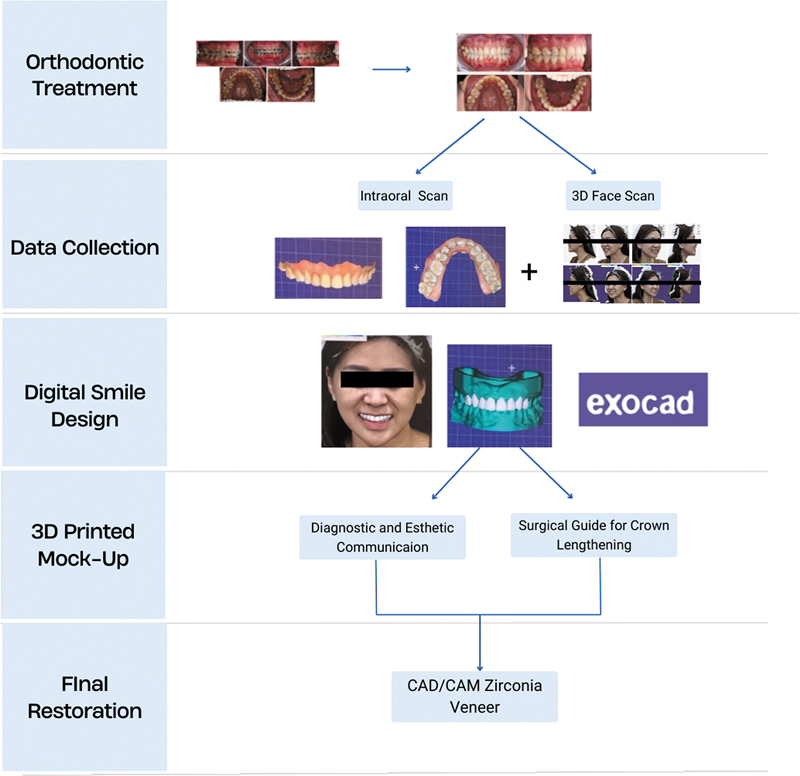
Illustration of the digital workflow in case management.

During the final appointment, the try-in of the zirconia CAD/CAM crowns was conducted in the patient's mouth, followed by interocclusal adjustment and assessment of canine guidance as well as protrusive and lateral movements, prior to glazing. The cementation of the glazed zirconia crowns was performed using dual-cure resin cement. The posttreatment frontal smile photograph showed a significant improvement in dental midline positioning, where the midline has been realigned toward the green zone, defined as the direction consistent with the patient's facial flow line. This realignment reflects a more harmonious integration between the dental and facial midlines, contributing to a more balanced and symmetrical appearance of the smile. As a result, the corrected midline not only enhances the visual coherence of the dentofacial structures but also reinforces the esthetic perception of facial symmetry and smile harmony ( Fig. 7 ). The digital restorative workflow is illustrated in Fig. 8 .

While the orthodontic treatment successfully addressed the dental midline deviation and occlusal concerns, the patient opted for veneers to further enhance the esthetic quality of her smile. The veneers were chosen to give the patient a more durable, precise, and customized esthetic result that met their expectations for a polished and natural-looking smile. This was especially important because the patient already had overcontoured veneers that did not fit well. The veneers were the last step in making the perfect smile that fit with the patient's face and overall dental health. Subsequent to all the procedures, a removable Essix retainer is positioned to maintain the stability of the orthodontic outcomes.

## Discussion

The successful outcome of this case emphasizes the importance of an interdisciplinary digital approach in esthetic rehabilitation postorthodontic therapy. Despite achieving orthodontic alignment, the patient had esthetic issues that orthodontics alone could not resolve. Orthodontic therapy is gradually developed as an important phase in a comprehensive esthetic rehabilitation process, wherein the use of digital technologies allows clinicians to establish outcomes based on entire facial harmony rather than solely on dental parameters. 11 12

In the current case, smile redesign was initiated through a 3D facial scan and intraoral scanner. To operationalize this into a restorative plan, digital smile design was performed using Exocad software. By integrating 3D facial scan data with intraoral scans, clinicians were able to create a facially guided virtual model that allowed real-time modifications of tooth proportion, display, and gingival display. This approach corresponds to macroesthetic concepts for facially driven treatment planning, aiming for natural esthetic harmony by coordinating dental structures with facial symmetry, lip mobility, and soft tissue dynamics. 6 11

Despite achieving satisfactory occlusal alignment through orthodontic treatment, some esthetic limitations persisted, particularly involving tooth proportion. Crown lengthening was performed on teeth 14 to 24 in this patient to facilitate veneer installation. The crown lengthening procedure is predicated on two fundamental principles: the establishment of biological width and the maintenance of sufficient keratinized gingiva around the tooth. 13 In this instance, a temporary crown was employed as both a diagnostic and esthetic guide to facilitate crown lengthening. Before the crown lengthening procedure, the temporary crown was fabricated in advance using a dental design software program to evaluate and mark the ideal gingival contour in accordance with the intended final smile design. As described in the previous report, using a temporary crown placed over the teeth to guide the desired contour through crown lengthening can help improve visualization and surgical precision and reduce procedure time, resulting in stable esthetic outcomes over long-term follow-up. 14 15

The integration of 3D-printed temporary materials has further streamlined the digital restorative workflow. Recent studies have shown that 3D-printed resins exhibit favorable surface roughness, superior microhardness, and acceptable color stability compared with traditional bisacrylic and acrylic resins, while maintaining full compatibility with digital protocols. 16 These materials allow clinicians to produce temporaries with better dimensional accuracy and surface characteristics, enhancing tissue compatibility and improving patient satisfaction during the provisional phase. 17

The digital veneer procedure starts with 3D digital smile design and virtual mock-up creation, followed by 3D printing of the virtual mock-ups to encourage patient treatment acceptance, and concludes with a chairside preparation guide aimed at optimizing enamel conservation during preparation. This protocol allows for the superimposition of mock-up scans and prepared teeth scans, ensuring that the final restoration faithfully replicates the planned esthetics while minimizing invasiveness and maximizing restorative fit. 18 19 In this case, teeth #14 and #24 were morphologically modified with veneer to substitute missing canine. This approach was chosen to restore both esthetic and functional guidance within the smile arc, particularly in the absence of natural canines (teeth #13 and #23).

CAD/CAM-fabricated monolithic zirconia veneers offer excellent biocompatibility, stable esthetics, and low abrasiveness to opposing dentition. 20 21 Designed at minimal thicknesses (0.2–0.3 mm), they enable conservative tooth preparation with precise marginal adaptation, often eliminating the need for subgingival finishing. Integrating these restorations into a fully digital workflow improves mechanical and esthetic outcomes while streamlining laboratory procedures without compromising clinical accuracy. 21 22 Following favorable orthodontic treatment, teeth are susceptible to relapse to their initial positions without a retention period. 23 A removable Essix retainer was invented thereafter to avert relapse. The Essix was utilized nightly for 12 months, thereafter 3 to 4 nights per week. Follow-up assessments at 3, 6, and 12 months evaluated midline stability, space management, occlusion, periodontal health, and prosthesis hygiene.

The integration of CAD/CAM technology and digital smile design was valuable, but making the digital workflow even better—especially when it comes to intraoral scanning, 3D smile design, and real-time digital adjustments—could lead to even more accurate results. Furthermore, further investigation into the long-term stability and adaptability of 3D-printed temporaries could enhance tissue compatibility and the patient experience.

## Conclusion

This clinical case underscores the effectiveness of a digitally guided interdisciplinary approach in postorthodontic esthetic rehabilitation. By integrating 3D facial scanning, intraoral scanning, digital smile design, and CAD/CAM-fabricated zirconia veneers, precise planning and execution were achieved to create a harmonious smile that integrates both facial and dental parameters. The use of a temporary crown as a diagnostic and surgical guide allowed for accurate gingival contouring during crown lengthening, while 3D-printed temporaries enhanced provisional esthetics and facilitated improved patient communication.

The fully digital workflow facilitated predictable esthetic outcomes, conservative tooth preparation, and enhanced functional and visual integration. This approach exemplifies the evolving role of digital dentistry in delivering individualized, minimally invasive treatments in complex interdisciplinary cases. Future research could investigate the feasibility of incorporating artificial intelligence into smile design to improve precision and customization.
